# How often does music and rhythm improve patients’ perception of motor symptoms in Parkinson’s disease?

**DOI:** 10.1007/s00415-013-6860-z

**Published:** 2013-02-12

**Authors:** C. Nombela, C. L. Rae, J. A. Grahn, R. A. Barker, A. M. Owen, J. B. Rowe

**Affiliations:** 1 Department of Clinical Neurosciences, Cambridge University, Cambridge, UK; 2 MRC-Cognition and Brain Sciences Unit, 15 Chaucer Road, Cambridge, UK; 3 Brain and Mind Institute, University of Western Ontario, London, ON Canada; 4 Department of Psychology, University of Western Ontario, London, ON Canada

Dear Sirs,

There is a strong interest in combining pharmacological treatments with non-drug therapies for Parkinson’s disease (PD). One such non-pharmacological therapy is music and rhythm stimulation, with anecdotal benefits and favorable preliminary clinical studies. It is suggested that the rhythmic properties of music reduce certain motor features of PD [1], perhaps by entraining the brain mechanisms that control timing, sequencing and coordination of movements [2]. Early investigations into the effect of auditory rhythms on movement and PD were promising [3], and their benefits and possible modes of action confirmed by recent neuroimaging studies [4]. However, we noticed an apparent discrepancy between the evolving literature on music, rhythm and PD, and the frequency of spontaneously reported benefits of music from patients in the clinic. We therefore asked: how commonly do patients themselves perceive an improvement in their motor symptoms with music and rhythm?

We designed and administered a written structured music questionnaire (Supplementary Material) to 50 patients with idiopathic PD (age 65.47 ± 7.8, stage I–III H&Y) during their routine visit to the Cambridge University PD Research Clinic. In addition, eight members of a PD choir (age 68.84 ± 6.9, II–III H&Y) completed the questionnaire, providing a highly motivated and musically experienced patient group. Participants completed the questionnaire (with multiple choice and open-ended questions) sitting in a quiet room. They were asked about (1) handedness, (2) symptomatic hearing problems (Y/N/details), (3) enjoyment of music (Y/N), (4) hours spent weekly listening to music, (5) styles of music listened to (free text), (6) formal musical training (instrument, type and years of training) and dance training (style, type and years of training), (7) current weekly time playing/performing music (Y/N/hours), and (8) whether they had ever noticed a beneficial effect of music on their PD symptoms (Y/N/free text details). Statistical analysis used IBM SPSS Statistics Version 19.

Of the clinic patients, 46 (92 %) reported normal hearing and 47/50 (94 %) enjoyed listening to music. Sixteen out of 50 (32 %) had formal musical training (average 5 years). Previous dance training was less frequent (10/50, 20 %) and of a shorter duration (average 3.2 years).

The entire clinic sample (100 %) reported no change in their PD symptoms when listening to music, although 32/50 (64 %) reported pleasant calm feelings. Therefore, despite the common benefit of experience relaxation while listening to music, there were surprisingly no perceived improvements in PD symptoms in this randomly selected cohort of patients.

Of the choir sample, four patients (50 %) had received musical training (average 5.2 years) and none had previous dance training. Apart from pleasure and a rewarding effect (in 7/8 patients), six patients reported no subjective beneficial effects from listening to music on their Parkinsonian symptoms. Two patients reported a general amelioration of their symptoms with a reduction of tremor.

This study provides preliminary evidence that a subjective beneficial effect of music on PD symptoms is uncommon. Although there is evidence for music stimulation as a potential therapy from clinical [2, 3] and neuroimaging [4] studies, it appears that patients with mild to moderate PD are often not aware of any significant effects on their motor function from listening to music. This largely negative result could be due to the insensitivity of our questionnaire, or it may be that the lack of such a subjective benefit is due to poor memory or awareness of actual benefits. Contributing factors may be the cognitive deficits that can arise even in early stages of PD [5], or inaccurate perception of movement [6].

Our data do not of course indicate a lack of objective benefit of music on motor signs. However, the low rate of positive responses (2/58 patients) poses a challenge for therapeutic studies. Music and rhythm therapies may need to train subjects to be more aware of their own state, or to target therapies at the subgroup of patients who have experienced musical benefit.

## Electronic supplementary material

Below is the link to the electronic supplementary material.

Supplementary material 1 (DOC 27 kb)
