# Supplementary material for: How often does music and rhythm improve patients’ perception of motor symptoms in Parkinson’s disease?
Source: J Neurol. 2013 Feb 12;260(5):1404–5. doi: 10.1007/s00415-013-6860-z (PMC3642358; doi:10.1007/s00415-013-6860-z)
Supplement: Supplementary file 1 — Supplementary material 1 (DOC 27 kb) [file 415_2013_6860_MOESM1_ESM.doc]

**For Experimenter Use Only:**

Participant # __________ Date ____________ Experiment ___________ Time ____________

# Music Questionnaire

1) Do you have normal hearing? Yes No

If not, please describe…………………………………………………………………………..

2) Do you enjoy listening to music? Yes No

3) What types of music do you enjoy, if any? …………………………………………………………...

…………………………………………………………………………………………………………..

4) Do you have any formal music training (for either voice or an instrument)? Yes No

If yes, which instrument(s) ………………………………………………………………………..

Please list the number of years for each instrument above………………………………………...

What type of training did you receive?

___School/Band ___Friends/Family

___Private Lessons ___Self Taught

___Church ___Other (Please explain)

5) Are you currently studying and/or performing music? Yes No

6) Do you have any formal dance training? Yes No

If yes, what style(s)? ……………………………………………………………………………

Please list the number of years for each style …………………………………………………..

What type of training did you receive?

___School ___Friends/Family

___Private Lessons ___Self Taught

___Other (Please explain)

7) Are you currently studying and/or performing dance? Yes No

8) Have you ever noticed a change in your symptoms when listening to music? Yes No

If yes, what symptoms changed, and how did they change? ……………………………………. ………………………………………………………………………………………………… ………………………………………………………………………………………………

………………………………………………………………………………………………

(Music Questionnaire v1.0, 24/2/2010)
